# A protocol to identify the barriers and facilitators for people with severe mental illness and/or learning disabilities for PErson Centred Cancer Screening Services (PECCS)

**DOI:** 10.1371/journal.pone.0278238

**Published:** 2022-11-30

**Authors:** Kate Sykes, Emma Tuschick, Emma L. Giles, Kehinde K. Kanmodi, Jill Barker

**Affiliations:** 1 Northumbria University, Newcastle upon Tyne, United Kingdom; 2 Teesside University, Middlesbrough, United Kingdom; PLoS ONE, UNITED STATES

## Abstract

**Objectives:**

To identify the barriers and facilitators that people with severe mental illness and people with learning disabilities may encounter when accessing cancer screening and make recommendations for implementing reasonable adjustments throughout cancer screening services.

**Methods and analysis:**

An 18-month sequential, mixed-methods study comprising of two phases of work and underpinned by Normalisation Process Theory, recruiting from across the North-East and North Cumbria. The first phase aims to identify the barriers and facilitators for people with severe mental illness in accessing cervical, breast and colorectal cancer screening. A systematic review of eight databases (Part 1a; PROSPERO registration number: CRD42022331781) alongside semi-structured interviews of up to 36 people with severe mental illness (Part 1b) will occur. Additional characteristics indicating populations whose perspectives may not have been accounted for in the systematic review will be targeted in the interviews. Potential participants will be identified from a range of settings across the North-East and North Cumbria, including through social media and gatekeepers within National Health Service Trusts and charities. Interviews will be analysed using framework analysis, which will be in line with the Normalisation Process Theory. The second phase of the project (part 2a) involves triangulating the results of the systematic review and interviews with existing research previously completed with people with learning disabilities accessing cancer screening. This will be to identify population specific barriers and facilitators across people with learning disabilities and people with severe mental illness to access cancer screening services. Following triangulation, part 2b will include designing and planning a future study involving stakeholders in cancer screening to explore the feasibility, practicality, and priority for implementing the recommendations to improve person centred cancer screening services (PECCS).

**Ethics and dissemination:**

This study has received Teesside University ethical approval, Health Research Authority approval (IRAS: 310622) and favourable opinion (REF: 22/PR/0793). Findings will be disseminated through a range of academic and non-academic modes including infographics, blog posts and academic publications.

## Introduction

Research suggests that the occurrence of most cancers is similar amongst People with Severe Mental Illness (PwSMI), People with Learning Disabilities (PwLD) and those without [1]. However, PwSMI and/or PwLD tend to die 15–20 years earlier than the general population [1]. Of these deaths, it is estimated that two out of three are from preventable physical illnesses, with higher rates of premature mortality in the North of England [2].

There are three cancer types that have an established screening programme in England; cancers of the cervix, breast, and colon [3]. Statistics show that PwSMI and PwLD are less likely to attend cancer screening than the general population [2]. Between 2020 to 2021 it was found that 32.7% of women with a learning disability attended breast cancer screening, compared to 65.4% of the general population [4], with the cancer screening uptake rates of PwSMI being substantially lower (by 40%) than the general population in England [5]. These statistics indicate significant inequalities in cancer screening access [1].

Previous systematic reviews and findings [2, 6] have identified barriers (for example, diagnostic overshadowing, fear, pain) and facilitators (for example, having information in an easy read format, desensitisation appointments) that can impact on attendance at cancer screening for both groups. However, there are gaps in current research that do not fully explore the factors influencing decision making and uptake of PwSMI, as well as identifying common factors across PwSMI and PwLD. This is important to understand given mortality rates in PwSMI and PwLD are continuing to rise in England by 5–10% every two years, and interventions are needed to address this [2]. The National Health Service (NHS) Long Term Plan and the government policy programme ‘Levelling Up’ white paper are both aimed at looking at increasing healthy life expectancy of up to five years by 2035 [7–9]. This would include promoting wellbeing, intervening earlier, improving medical pathways for PwSMI, and addressing disparities [7]. Additionally, The Equality Act 2010 is a legal framework enabling people to access services in the UK, including healthcare services [10]. Within the Act, it is emphasised that reasonable adjustments must be made where it is reasonable to do so. The identification of the barriers and facilitators to support decision making and access to screening, fall under the implementation of reasonable adjustments to ensure everyone has equal access to the healthcare setting or service.

Whilst there has been similar research undertaken in this area [11–13], this research will explore the current gaps in the literature and then fill this evidence gap. It is important that we identify reasonable adjustments that can be embedded in the cancer screening services to support the uptake of PwSMI and/or LD. By exploring the barriers and facilitators within the three cancer screening services, this will enable us to make relevant, well-informed, and practical recommendations for improvements to enhance person centred care. Person centred care is a model where healthcare providers are encouraged to partner with patients, co-design, and deliver personalised care that provides people with the high-quality care they need and improve health-care system efficiency and effectiveness [14].

### Aims and objectives

The overall aim is to identify reasonable adjustments that can be embedded throughout cancer screening services to ensure person centred screening services, which may support uptake and participation by PwSMI and/or PwLD.

To achieve this aim, three substantive objectives have been set:

1. To explore the barriers and facilitators for PwSMI in accessing NHS cancer screening services.
2. To make recommendations for healthcare practice, ensuring equality of access that is person centred for PwSMI and/or PwLD to cancer screening services.
3. To plan and design future research to obtain an expert consensus on solutions to implement recommendations to support informed and shared decision making and uptake of cervical, breast and colorectal cancer screening for PwSMI and PwLD.

## Methods

### Study design

**Fig 1:**
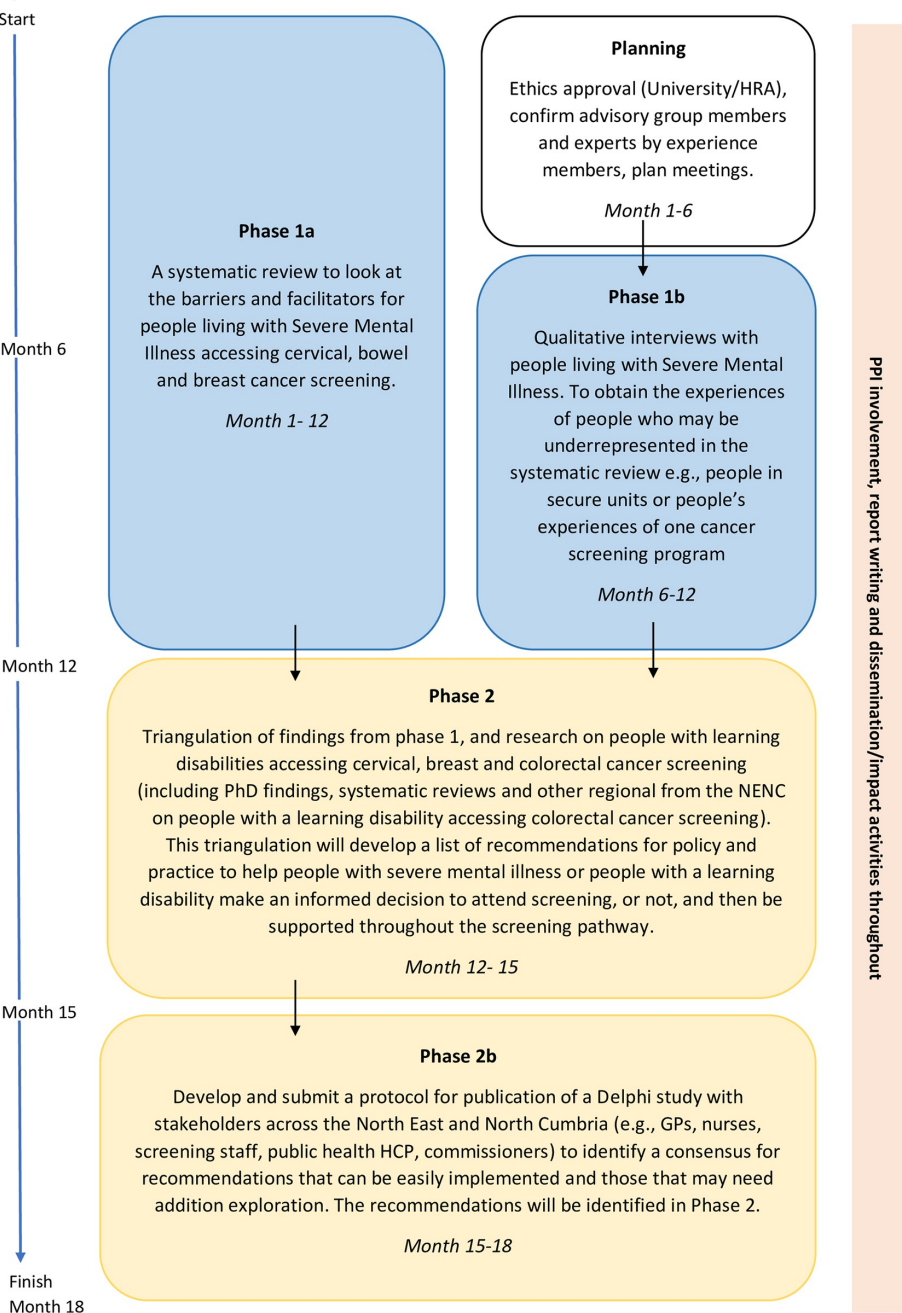
Flowchart.

This is a mixed method study, to be completed over 18 months from March 2022 to September 2023, consisting of two phases (see Fig 1):

- Phase one: Part 1a) a systematic literature review of barriers and facilitators to cancer screening for people with severe mental illness (PwSMI); Part 1b) qualitative interviews with PwSMI.
- Phase two: Part 2a) triangulation of research findings; Part 2b) planning and designing future research.

### Theoretical underpinning

**Fig 2:**
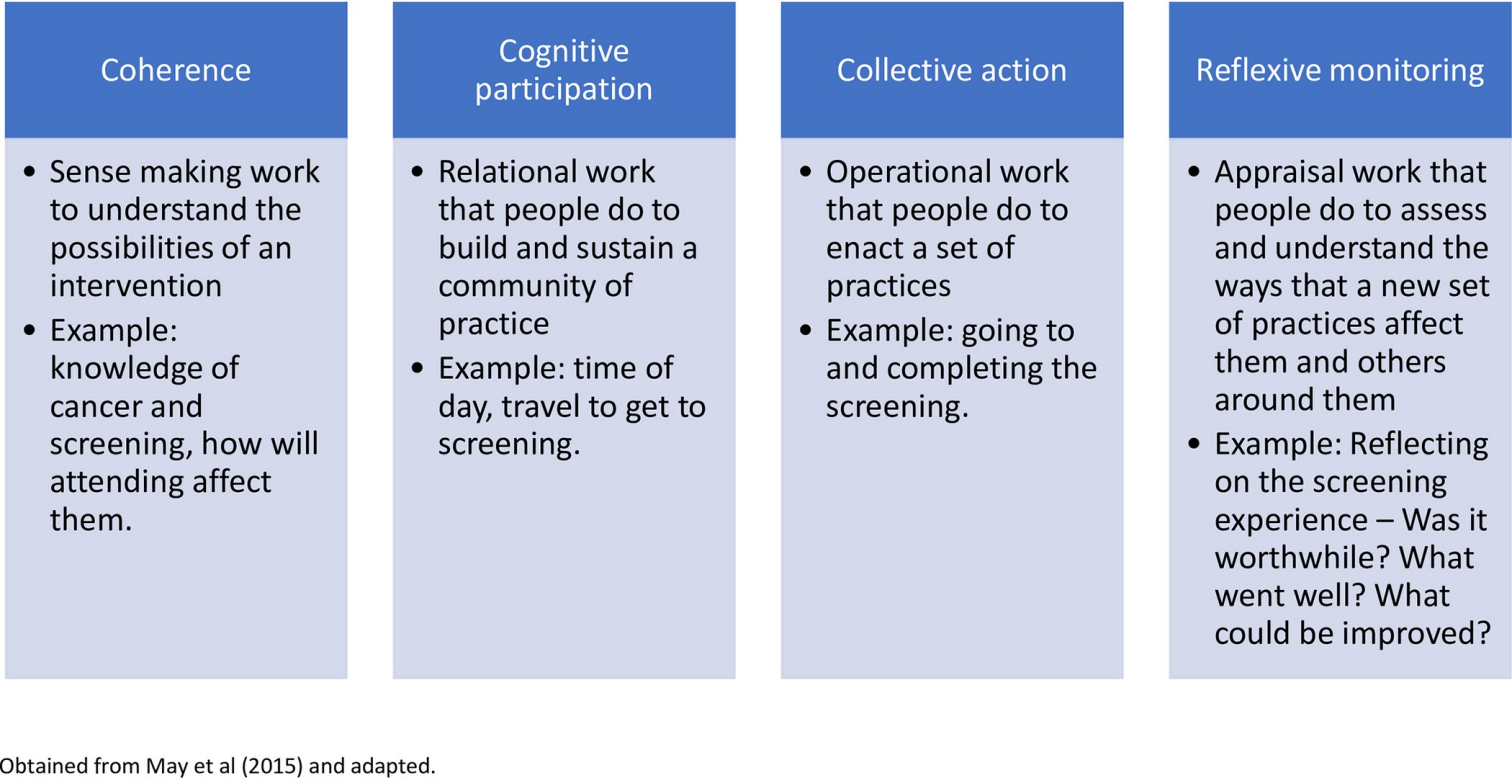
Constructs of NPT.

Normalisation Process Theory (NPT) (see Fig 2) is an implementation theory for understanding how interventions are implemented to become part of normal practice, with a specific focus on the work that people and groups do. NPT will underpin all phases of this study [15, 16]. The theory is comprised of four constructs: coherence, cognitive participation, collective action, and reflexive monitoring [17]. A systematic review of 108 studies using NPT identified it accurately illustrated important elements of implementation processes [18]. Additionally, the review found that NPT can effectively explain the successes or failures of specific implementation projects [18]. Therefore, NPT was chosen for this study to ensure the research is focused on implementation science to minimise the barriers and enhance the facilitators to ensure access to and acceptability of cancer screening.

### Phase 1

Phase 1 will consist of two parts: (1a) a systematic review of the barriers and facilitators to PwSMI accessing cancer screening, and (1b) semi-structured qualitative interviews with PwSMI. This phase will address study objective 1 (to explore the barriers and facilitators for PwSMI in accessing NHS cancer screening services).

#### Part 1a: Systematic literature review

The aim of the review is to synthesise the evidence of the barriers and facilitators on PwSMI accessing cervical, breast or colorectal cancer screening. The review will not include PwLD, as two published systematic reviews already address this [19, 20]. The review is registered on PROSPERO (CRD42022331781) and will be reported in line with the Preferred Reporting Items for Systematic Reviews and Meta-Analyses Protocols (PRISMA-P) [21] (S1 File). The protocol for the review has been written in consultation with an academic librarian to ensure the rigour of the search strategy.

*Database searches and management*. PsycINFO, MEDLINE, SCOPUS, CINAHL, Embase, ProQuest Nursing & Allied Health will be searched, along with MEDNAR and Google Scholar, where the first 100 hits will be retrieved. The search string can be seen in S2 File. Searches will be conducted in line with the Population, Issue, Outcome and Study (PIOs) framework [22]: Population relates to PwSMI, which we define as “individuals who have received a diagnosis of schizophrenia or bipolar affective disorder, or who have experienced an episode of non-organic psychosis” [23], as well as carers and/or healthcare professionals; Issue relates to cancer screening; Outcome relates to barriers/facilitators/experiences/knowledge and Study relates to any empirical research from any county. All results will be downloaded into EndNote (Clarivate Analytics, V.X9), where duplicate records will be removed prior to the sifting.

Title and abstract sifting will be completed by one reviewer. Two reviewers will independently double screen 20% each. Each reviewer will document their screening decisions on a separate Microsoft Excel spreadsheet. If discrepancies are identified, they will be discussed between the reviewers to make a final decision on whether the paper is potentially relevant and should go through to the second sift. If a decision cannot be made, an independent third reviewer will decide. Following titles and abstract sift, full paper screening will take please with double screening being completed on all potentially eligible papers. Again, each reviewer will document their screening decisions on a separate Microsoft Excel spreadsheet. The reference lists of included papers will be searched for any additional outputs that would be eligible.

*Inclusion criteria*. To determine eligibility of returned papers, inclusion criteria will be applied that are based on the PIOs framework [22]. Papers will not be limited by location, year of publication or research method used but will be limited to those written in English, focus on cervical, breast or colorectal cancer, with a focus on PwSMI, and/or their carers and healthcare professionals.

*Analysis*. Dixon-Woods framework-based synthesis [24] will be used for the data analysis. This framework was chosen as it will allow the analysis to conform to the concepts of NPT. As this study will be guided by NPT, this is considered the ‘best fit’ strategy for when it comes to implementing and making changes to the cancer screening services [24]. To ensure the data is synthesised in line with NPT, a Microsoft Excel spreadsheet will be developed prior to data extraction. The spreadsheet will capture; the authors, year of publication, country of study, aim of the research, study design, methods, setting/location of research, sample size, participant demographics, quality of study, data about sense making, enrolment, enactment and appraisal, and any non-NPT data that relates to the topic. The mixed methods appraisal tool (MMAT) will be used for the quality assessment of the included articles.

#### Part 1b: Semi-structured Interviews

The aim of the qualitative interviews is to explore and fill any research gaps identified through the systematic review, of the barriers and facilitators of PwSMI towards cancer screening. These gaps may pertain to particular characteristics/features or contexts of PwSMI who are not represented in the systematic review.

*Participants and sampling*. In line with Braun and Clarke’s recommendations for completing thematic analysis [25], we will aim to recruit a maximum of 12 people per group (three groups in total). This will give an upper recruitment target of 36 PwSMI. The Projects Advisory Board (PAB) will determine the three groups to be prioritised, these may reflect regional priority groups.

Through purposive sampling all eligible participants must meet the following inclusion criteria: age 25 years or over; live in the North-East of England and North Cumbria; can consent to participate; and be able to speak and understand English. As the three groups are to be determined following the gaps in evidence from the systematic review, specific inclusion criteria will be developed and added to the list. For example, the groups may focus on different specific SMI, or cancer screening types that may be under-represented in the systematic review.

*Recruitment strategy*. Participants will be recruited from across the North-East and North Cumbria integrated care system with the support of a range of gatekeepers within NHS acute, mental health and primary care services, and voluntary and charitable sector organisations. Gatekeepers will provide information about the study to those who meet the inclusion criteria. If individuals meet the criteria, the gatekeeper will contact them using a person-centred approach, for example by using their preferred method of contact (such as in the next appointment, or over the phone). Gatekeepers will describe the study and if the potential participants want to hear more about the study, they can either: 1) provide verbal consent to be contacted by the researcher, or 2) make direct contact with the researcher themselves using the contact details on the participant information sheet. If option 1, the gatekeeper or clinicians will complete a contact slip with the person contact details. This will be passed onto the research in person, or electronically through a password protected file. The contact slip will document the name, address, contact information and their preferred method of contact. If the person is unsure, the gatekeeper will contact them one week later to confirm their decision. If the potential participant does not wish to participate, then no further contact will be made.

The data from the contact form will be input into a password protected excel document Participants will then be contacted by the research team using the participants preferred method of contact. If the participant still wishes to take part, an appointment will be made for an interview at a time and location of the participants choice, this could be face-to-face, over the telephone or via Microsoft Teams and will be confirmed by email or letter. The Participant Information Sheet (PIS) and consent form will then be sent to the participants to read over, and they will be given the opportunity to ask any questions. The PIS has been co-produced with experts by experience of severe mental illness working with the research team to ensure this is in a jargon free format, using plain English.

The study will also be advertised through social media, for example Twitter and Facebook. Specific social media groups, such as charities, will be targeted to ensure recruitment is more precise and controlled as these specific groups on this platform are administered by organisations with the group members having similar interests (and could potentially be more open to sensitive discussions). We will verify their status from social media by having a discussion with them before the interview stage to establish their condition. This will be a ‘self-declared’ diagnosis with the definitions being initially discussed to meet the inclusion criteria at an early stage.

The social media materials have also been co-produced to be inclusive using clear text, using the same colours, voice-overs on Tweets, and alt text on images so that communications are as accessible as possible. If a person meets the inclusion criteria, and has seen the study advertised on social media, they can contact the researcher directly (whose email is provided on the poster), or they can complete an expression of interest form (an electronic set of questions that people complete held on the JISC platform). The researcher can then contact the person directly using the information provided. The question on the expression of interest form includes: the person’s name, the first part of their postcode, how did they hear about the study, their preferred method of contact, and the best contact details for the person, for example their email address or phone number.

*Data collection*. Semi-structured, one-to-one interviews will be conducted in line with the participants choice of either face-to-face (at a chosen location by the participant), over the phone or over Microsoft Teams. Informed consent will be sought prior to the interview, this will be written consent for face-to-face interviews, or verbal consent for interviews conducted over the phone or via teams.

A demographic questionnaire will firstly be given to the participants to complete on a JISC platform on the researcher’s laptop. The questionnaire will ask questions based around the participants age, gender, education and lifestyle and will consist of 13 questions. The interview questions will be designed to gain a deeper understanding of participants’ experiences to implement future change by exploring barriers and facilitators that can then highlight issues of implementation, embedding, and integration reasonable adjustments within the screening programmes. The interview schedule will be coproduced with the experts-by-experience, ensuring questions are appropriate and help to answer the research questions from a service user’s point of view. The interviews will not last longer than 60 minutes each.

*Data analysis*. Data will be analysed by members of the research team and experts by experience using framework analysis which is line with the NPT constructs (coherence, cognitive participation, collective action, reflective monitoring). There are multiple benefits of using a framework analysis that are in line with NPT, including; 1) it provides an explicit audit trail of how the findings were developed [26]; and 2) the analysis may provide a direct, theory-driven structure to assess the implementation of interventions [27].

*Data management and security*. Data management and security measures align with the Health Research Authority [28] and Teesside University guidelines [29]. The audio files of the interviews will be uploaded onto the universities secure immediately after the interview and deleted from the Dictaphone. All participants will be reminded that their participation is completely voluntary and all information that they provide will be kept securely. All personal and identifiable information will be removed and replaced with a unique participant number. One password protected spreadsheet will be created linking the participants contact names and their allocated unique participant number. The linking document will be destroyed when data collection has been completed. Only the research team will have access to the audio file and transcripts. All participants can be given a copy of the transcript if they wish.

### Phase 2

Phase 2 will comprise of two components: (1) the triangulation of research identified in Phase 1 with previous research involving PwLD going to cancer screening, and (2) planning and designing future research. This phase will address study objectives 1 and 2.

#### Part 2a: Triangulating WP1 with previous research

The data and results obtained from parts 1a and 1b will be triangulated with the findings from previous research that focuses on cancer screening attendance by PwLD [6, 30–32]. This research on PwLD discovered the barriers to cancer screening included embarrassment, unpreparedness, negative interactions with healthcare professionals, a lack of knowledge, and fear [30]. Facilitators that were identified included living in a supervised setting, prior use of other healthcare services, and being educated about screening via social media [30].

This triangulation will develop a set of recommendations for policy and practice to improve the uptake of cancer screening of PwSMI and PwLD, which will subsequently be used the planning and design of a Delphi study (part 2b). Farmer’s [33] triangulation protocol will be used to complete the triangulation work. This includes following the 6-step procedure: sorting, convergence coding, convergence assessment, completeness comparison, researcher comparison and feedback [33]. By undertaking this triangulation protocol (which is well suited to mixed method research), this will enable the study to bring together multiple perspectives on the research question and will largely add to the studies validity [34]. The triangulation will be undertaken and completed by the research team and members of the PAB following the above 6-step procedure. A document will be developed highlighting the aspect of NPT, the subcategories, and definitions for each. The triangulation will identify common barriers and facilitators across PwSMI and PwLD, as well as population specific consideration, in accessing cancer screening. These will be used to develop recommendations (in line with NPT) for policy and the screening programmes. The triangulation will be completed in a workshop with the research team and members of the PAB (either face-to-face, via Microsoft teams, or a hybrid approach of the two). Experts by Experience will support the discussions and interpretation of the triangulated results and recommendations.

#### Part 2b: Planning and designing future research

We will use the findings from 2a to write a protocol for future research, which will form a Delphi study. This will be used as the basis of a future research bid. The Delphi process is a structured group facilitation technique to obtain consensus among anonymous respondents. The aim of the Delphi is to gain a consensus from experts within cancer screening services (e.g., mammogram staff, cervical sample takers, commissioners, GPs) across the NENC region, to identify the recommendation that can be implemented in the short-, medium- and long-term future, as well as identifying barriers and facilitators to implementing the recommendations. As a result of this, the engagement of stakeholders may increase the ownership of changes and recommendations for practice, as well as improving the population’s participation and the research outcomes and moral grounds [35]. This can then aid in employing reasonable adjustments within the cancer screening services to be more person centred, accessible and acceptable for PwSMI and/or PwLD.

### Ethical considerations

The project will adhere to the UK Policy Framework for Health and Social Care Research and will comply with the General Data Protection Regulation and Data Protection Act [20]. The study has received ethical approval from a University, Health Research Authority (HRA) approval IRAS Ref: 310622) and favourable opinion from a HRA Research Ethics Committee (ref: 22/PR/0793).

### Patient and public involvement

The National Institute for Health and Care Research (NIHR) [36] encourages studies to include patient and public involvement (PPI) as this ensures the study is providing a different perspective, making the research more relevant and overall improving the quality of the research. As part of PPI, NIHR recommends the following areas of research have active participation by the PPI members, including designing and managing, undertaking, disseminating and implementing. By having PPI, this allows our study to fully engage and promote NIHR’s ‘Standards’, including, inclusive opportunities (important for our study as PwSMI are underrepresented in this area of research), working together (valuing other people’s contributions), support and learning (building confidence in others), communications (identifying specific communication needs amongst the population), impact (sharing our combined knowledge) and governance (allow PPI members to make decisions).

PPI plays a major part of our research and runs throughout the study. An Experts by Experience Advisory Group (EbEAG) has been set up comprising of three PwSMI (currently employed) and three PwLD (to be recruited for the second phase of the study) to support the research. The experts by experience have helped in a range of ways, including; the recruitment of a research associate, in the development of recruitment material for phase 1b, and advised on ethical queries. Moving forward, they will be engaged in the design, management, analysis, evaluation, and dissemination of research outputs. Co-production has been achieved by involving PwSMI in sense checking the systematic review search strategy and co-designing the participant information sheet and interview schedule. Experts by Experience will be involved in triangulating findings, developing recommendations for practice and the dissemination of findings. Throughout the project, the EbEAG will meet regularly with the research team, and be invited to attend the PAB. A table has been developed (S3 File), based on the GRIPP2 short form [37], which captures the task, aims, methods, results, discussions and reflections of all our communications with the experts by experience. By following this model, we aim to improve the quality, transparency, and consistency of our PPI to ensure optimal results. Lastly, we hope to build and maintain the relationships we develop with our PPI members, allowing for personal growth and development, as well as continuous reflection amongst the research team.

#### Project advisory board

The PAB is formed of members from organisations across the ICS including: NHS organisations, General Practices, the voluntary and charitable sector, universities, the cancer screening programmes and PPI members. The PAB will ensure clinicians in primary care and secondary care, within services for PwSMI and PwLD are brought together to advise on the research. This may then enhance or build new partnership working between services in the region, to account for the needs of PwSMI and PwLD. The PAB will meet regularly throughout the research timeline.

#### Dissemination

Throughout the course of the research, dissemination of the findings will occur and will be published on the study website (https://hosting.northumbria.ac.uk/peccs/). The dissemination of research has been divided into a short-, medium- and long-term impact plan (S4 File). To summarise, the study aims to disseminate the research findings through academic and non-academic methods. This will include, but not limited to, presentations at conferences, academic papers, blog posts and the development of infographics. The dissemination of the research will be supported and guided by the EbEAG.

## Discussion

This study is unique in that it aims to identify current barriers to cancer screening access and conceptualises them into real-world applicable solutions to help a seldom heard population. Firstly, this study will be reviewing all available literature surrounding cancer screening for PwSMI, identifying gaps, and fully immersing itself in the current barriers and facilitators they hold. Alongside this, this study is benefited by having an Experts by Experience group, which enables the study to be fully engaged with the audience it is targeting. Secondly, semi-structured interviews will allow the study new insights into the current cancer screening services that are offered. This will help form recommendations, reasonable adjustments and procedural changes that are needed to create a ‘Person-Centred’ pathway for UK cancer screening services that are in line with the equality act and the ‘parity of esteem’ concept to reducing mortality rates by giving equal priority to physical health [38–41]. Thirdly, the triangulation will inform recommendations for practice and inform the three cancer screening services. Lastly, the Delphi study will involve stakeholders in cancer screening to come to a consensus on the ease of implementing the recommendation, the barriers to implementation, and future research priorities. To conclude, healthcare providers and cancer screening services will be able to use this research and its person-centred recommendations to improve cancer screening services and create a positive future impact for PwSMI and/or PwLD.

## Supporting information

S1 File PRISMA-P-checklist for systematic review. (DOC)

S2 File Search string for systematic review. (DOCX)

S3 File PPI summary table for interviews. (DOCX)

S4 File Dissemination. (DOCX)
