# Supplementary material for: A protocol to identify the barriers and facilitators for people with severe mental illness and/or learning disabilities for PErson Centred Cancer Screening Services (PECCS)
Source: PLoS One. 2022 Nov 30;17(11):e0278238. doi: 10.1371/journal.pone.0278238 (PMC9710752; doi:10.1371/journal.pone.0278238)
Supplement: S2 File — (DOCX) [file pone.0278238.s002.docx]

**Draft MEDLINE search v.2 4.5.22**

| **#** | **Query** | **Results** |
| --- | --- | --- |
| S1 | (MH "Schizophrenia Spectrum and Other Psychotic Disorders+") | 157,716 |
| S2 | (MH "Bipolar Disorder") | 43,535 |
| S3 | (serious OR severe) N2 (mental illness OR mental disorder) | 13,220 |
| S4 | psychosis OR psychoses OR psychotic OR paraphrenia OR schizophreni* OR bipolar OR "delusional ideation" OR (manic N2 depress*) OR PwSMI | 288,305 |
| S5 | S1 OR S2 OR S3 OR S4 | 298,123 |
| S6 | (MH "Embarrassment") | 66 |
| S7 | (MH "Emotions+") | 378,806 |
| S8 | (MH "Healthcare Disparities") | 20,934 |
| S9 | (MH "Health+") | 412,546 |
| S10 | (MH "Health Inequities+") | 18,934 |
| S11 | (MH "Socioeconomic Factors+") | 488,072 |
| S12 | (MH "Prejudice+") | 34,766 |
| S13 | (MH "Risk+") | 1,335,054 |
| S14 | (MH "Social Stigma") | 11,340 |
| S15 | (MH "Time+") | 1,407,345 |
| S16 | (MH "Uncertainty") | 15,760 |
| S17 | aggravat* OR barrier* OR burden* OR challeng* OR complex* OR confront* OR contest* OR defy* OR defiance OR difficult* OR disparit* OR embarrass* OR emotion* OR fear* OR health* OR hurdle* OR impediment OR impractical OR inequalit* OR intrusive OR issue* OR lack* OR mistrust* OR negative* OR object* OR obstacle OR obstruct* OR oppos* OR paranoi* OR prejudice OR psychosocial OR "psycho-social" OR question* OR remember* OR risk* OR "self-conscious*" OR selfconscious* OR stigma* OR therap* OR time OR traumat* OR inaccessibi* OR uncertainty OR unwell | 19,543,224 |
| S18 | (MH "Health Services Accessibility+") | 121,778 |
| S19 | (MH "Intersectoral Collaboration") | 2,554 |
| S20 | (MH "Communication+") | 344,542 |
| S21 | (MH "Treatment Adherence and Compliance+") | 268,029 |
| S22 | (MH "Continuity of Patient Care") | 20,327 |
| S23 | (MH "Knowledge") | 13,148 |
| S24 | (MH "Motivation+") | 185,863 |
| S25 | (MH "Health Promotion+") | 82,901 |
| S26 | (MH "Comprehension") | 16,479 |
| S27 | accessibil* OR accomodat* OR aid OR benefi* OR broadcaster OR broker OR catalyst OR collaborat* OR communicat* OR compliance OR conciliator OR confidence OR continuity OR coordinator OR "co-ordinator*" OR enabler OR encourag* OR expert* OR facilitat* OR "health-conscious*" OR help* OR inform* OR intermediary OR interven* OR knowledg* OR location OR mediator OR motivat* OR positiv* OR promot* OR relation* OR respons* OR strateg* OR support OR understand* OR willing* | 14,804,135 |
| S28 | (MH "No-Show Patients") | 232 |
| S29 | (MH "Attitude+") | 621,047 |
| S30 | (MH "Awareness") | 21,457 |
| S31 | (MH "Behavior+") | 1,996,308 |
| S32 | (MH "Culture+") | 173,623 |
| S33 | (MH "Consciousness") | 13,005 |
| S34 | (MH "Social Determinants of Health") | 5,449 |
| S35 | (MH "Social Discrimination+") | 10,726 |
| S36 | (MH "Self-Examination+") | 2,716 |
| S37 | (MH "Thinking+") | 308,542 |
| S38 | apprehension OR attendance OR attitud* OR awareness OR behaviour* OR behavior* OR beliefs OR consciousness OR delay* OR determinants OR discrimination OR experience OR factors OR feelings OR knowledge OR "non-attend*" OR nonattend* OR "non-participat*" OR nonparticipat* OR opinions OR participation OR perception OR problem OR realisation OR realization OR reasons OR recognition OR "self-examination*" OR thoughts OR uptake OR "up-take" or takeup OR "take-up" OR views OR enrol* | 11,950,210 |
| S39 | S6 OR S7 OR S8 OR S9 OR S10 OR S11 OR S12 OR S13 OR S14 OR S15 OR S16 OR S17 OR S18 OR S19 OR S20 OR S21 OR S22 OR S23 OR S24 OR S25 OR S26 OR S27 OR S28 OR S29 OR S30 OR S31 OR S32 OR S33 OR S34 OR S35 OR S36 OR S37 OR S38 | 25,153,603 |
| S40 | (MH "Breast Neoplasms+") | 324,293 |
| S41 | (MH "Colorectal Neoplasms+") | 223,755 |
| S42 | (MH "Uterine Cervical Neoplasms") | 81,314 |
| S43 | S40 OR S41 OR S42 | 615,620 |
| S44 | (MH "Early Detection of Cancer") | 33,167 |
| S45 | S43 AND S44 | 17,943 |
| S46 | (breast OR colorectal OR colon OR bowel OR cervi*) N5 (screen* OR assessment OR test* OR early detection OR early diagnos* OR periodic examination) | 81,467 |
| S47 | S45 OR S46 | 86,135 |
| S48 | (MH "Vaginal Smears") | 22,646 |
| S49 | (MH "Occult Blood") | 6,064 |
| S50 | (MH "Mammography+") | 32,114 |
| S51 | (screen* OR test*) N5 (smear OR pap OR Papanicolaou OR HPV OR mammogra* OR mastography OR fecal occult OR faecal occult OR fobt OR fob OR fecal immunochemical OR faecal immunochemical) | 39,979 |
| S52 | S48 OR S49 OR S50 OR S51 | 78,759 |
| S53 | S47 OR S52 | 136,113 |
| S54 | S5 AND S39 AND S53 | 117 |
| S55 | S5 AND S39 AND S53 Limiters - English Language | 113 |
