# Supplementary material for: A protocol to identify the barriers and facilitators for people with severe mental illness and/or learning disabilities for PErson Centred Cancer Screening Services (PECCS)
Source: PLoS One. 2022 Nov 30;17(11):e0278238. doi: 10.1371/journal.pone.0278238 (PMC9710752; doi:10.1371/journal.pone.0278238)
Supplement: S3 File — (DOCX) [file pone.0278238.s003.docx]

**PPI Summary Table**

**Key: (GRIPP2 short form)**

1: Date: Date of the entry

2: Task: What is asked of PPI

3: Aim: Report the aim of PPI in the study

4: Methods: Provide a clear description of the methods used for PPI in the study

5: Study results: Outcomes—Report the results of PPI in the study, including both positive and negative outcomes

6: Discussion and conclusions: Outcomes—Comment on the extent to which PPI influenced the study overall. Describe positive and negative effects

7: Reflections/critical perspective: Comment critically on the study, reflecting on the things that went well and those that did not, so others can learn from this experience

PPI=patient and public involvement

EBE=experts by experience

| **1.Date** | **2.Task** | **3.Aims** | **4.Methods** | **5.Results** | **6.Discusion** | **7.Reflections** |
| --- | --- | --- | --- | --- | --- | --- |
|  |  |  |  |  |  |  |
|  |  |  |  |  |  |  |
|  |  |  |  |  |  |  |
|  |  |  |  |  |  |  |
