# Supplementary material for: A protocol to identify the barriers and facilitators for people with severe mental illness and/or learning disabilities for PErson Centred Cancer Screening Services (PECCS)
Source: PLoS One. 2022 Nov 30;17(11):e0278238. doi: 10.1371/journal.pone.0278238 (PMC9710752; doi:10.1371/journal.pone.0278238)
Supplement: S4 File — (DOCX) [file pone.0278238.s004.docx]

**Dissemination**

Throughout the course of the research, dissemination of the findings will occur and will be published on the studies study website (https://hosting.northumbria.ac.uk/peccs/). The dissemination of research has been broken down into a short-, medium- and long-term impact plan.

**Short-Term**

- Systematic review (including; PROSPERO registered, conference abstracts, share findings in FUSE blog, present findings within university research seminars, disseminate on progress through social media platforms, infographic of barriers and facilitators).
- Triangulation of findings (including; NPT infographic of barriers and facilitators, flow diagram highlighting pathways to be embedded, write and share a blog on FUSE and Public Health England, Health inequalities blog)
- Delphi study protocol (including; academic research paper, share and disseminate via FUSE blog, disseminate on progress through social media platforms).

**Medium-Term**

- Systematic review (including; submit conference abstracts, collaborate with regional service users and experts by experience to share findings, engage with and present to regional networks and forums).
- Triangulation of findings (including; produce an academic research paper on triangulation, disseminate findings at Mental Health and Learning weeks at Universities, present findings to service user groups.)
- Delphi study protocol (including; secure additional research funding to complete the Delphi study).

**Long-Term**

- Systematic review (including; work in partnership with organisations across the North East & North Cumbria, identify learning from this project and how it could be applied to other national screening programmes that PwSMI may access).
- Triangulation of findings (including; produce guidance on supporting informed and shared decision making and access to screening for hard-to-reach groups, work with CCG’s to improve equality of access to cancer screening, collaborate with Integrated Care Boards (ICBs) to employ a person-centred approach to cancer screening using reasonable adjustments).
- Delphi study protocol (including; to complete the Delphi study and make recommendations for policy and practice).
